# Tunable tapered waveguide for efficient compression of light to graphene surface plasmons

**DOI:** 10.1038/srep28799

**Published:** 2016-06-29

**Authors:** Bo Han Cheng, Hong Wen Chen, Yi-Jun Jen, Yung-Chiang Lan, Din Ping Tsai

**Affiliations:** 1 Department of Electro-Optical Engineering, National Taipei University of Technology, Taipei 106, Taiwan; 2 Department of Photonics and Advanced Optoelectronic Technology Center, National Cheng Kung University, Taiwan 70101, Taiwan; 3 Research Center for Applied Sciences, Academia Sinica, Taipei 115, Taiwan; 4 Department of Physics, National Taiwan University, Taipei 10617, Taiwan

## Abstract

Dielectric-graphene-dielectric (DGD) structure has been widely used to construct optical devices at infrared region with features of small footprint and low-energy dissipation. The optical properties of graphene can be manipulated by changing its chemical potential by applying a biased voltage onto graphene. However, the excitation efficiency of surface wave on graphene by end-fire method is very low because of large wavevector mismatch between infrared light and surface wave. In this paper, a dielectric-semiconductor-dielectric (DSD) tapered waveguide with magnetic tunability for efficient excitation of surface waves on DGD at infrared region is proposed and analyzed. Efficient excitation of surface waves on DGD with various chemical potentials in graphene layer and incident frequencies can be attained by merely changing the external magnetic field applied onto the DSD tapered waveguide. The electromagnetic simulations verify the design of the proposed structure. More importantly, the constituent materials used in the proposed structure are available in nature. This work opens the door toward various applications in the field of using surface waves.

Surface plasmon polaritons (SPPs) on metal surface have attracted a lot of attention from all over the world for its promising optical property, such as supporting subwavelength surface wave^1^, breaking diffraction limit^2^,^3^,^4^,^5^, high extinction ratio^6^, and guiding light on the top of metal without the use of photonic band gap design. For key applications, such as bio-sensing^7^, negative refractive index^8^,^9^, metasurface^10^,^11^,^12^,^13^, and photocatalytic^14^,^15^, especially for the demand of high-density optoelectronic components^16^, it is crucial to have smaller footprint to perform the specific function, which is composed of rationally designed nanostructured building blocks. Generally, SPPs propagating on a continuous noble metal film at visible region can support surface wave with size of hundreds of nanometers^9^, while the extension length of electromagnetic field from the interface tends to be half of the wavelength of excited wave^9^, limiting the pursuit of future device with the mentioned task of ultrahigh density design.

The wavelength and extension length of surface waves can be reduced to dozens of nanometer under infrared illumination by replacing metal with two-dimensional material, graphene. Therefore, the use of graphene provides us a path to make an ultra-compact device^17^. Several useful optical devices based on graphene plasmonics have been investigated, such as tunable terahertz metasurface^18^, carbon nanotube-graphene resonator^19^, and active plasmonic devices^20^,^21^,^22^. Furthermore, the energy dissipation of the tightly confined surface wave on graphene is far smaller than that on metal^17^. If the surface waves on gaphene can be efficiently excited from the incident light, an optical device with features of small footprint and low-energy dissipation can be obtained.

There are many methods for launching the surface wave on graphene, such as prism coupling excitation^23^, electron beam excitation^24^, atomic force microscopy (AFM) tip based excitation^25^ and end-fire excitation method^26^. Compared to other methods, the end-fire excitation is widely adopted for efficient excitation of surface waves at a dielectric-metal interface due to the feature of compatibility and convenience. It is also the simplest method for launching electromagnetic waves in the waveguide connection. When the geometry optimization is conducted, more than 90% of coupling efficiency can be achieved^27^. However, the end-fire excitation is not efficient for directly exciting graphene’s surface wave due to large wavevector mismatch between infrared light in free space and surface wave on graphene. Recently, the efficient coupling of light into graphene surface waves by using prism coupler and tapered bulk materials has been proposed^23^.

In this work, a semiconductor tapered waveguide with magnetic tunability for efficient end-fire excitation of gaphene surface waves at infrared region for the demand of real application is proposed and analyzed. High coupling efficiency of excitation of graphene surface waves with different chemical potentials in graphene layer and different incident frequencies can be attained by merely changing the external magnetic field applied onto the tapered waveguide. Compared to previous investigations, this work focuses on adding another degree of freedom for manipulating the coupling efficiency between semiconductor surface waves and graphene surface waves by applying an external magnetic field.

Figure 1(a,b) plot the proposed indium antimonide (InSb)-based tapered waveguide and its side-view (in xy-plane), respectively. The tapered waveguide that consists of five dielectric-semiconductor-dielectric (DSD) elements (the semiconductor material is InSb) is placed in front of dielectric-graphene-dielectric (DGD) structure. (DSD and DGD use the same dielectric substrate). The relative permittivity of the substrate (i.e. the lower dielectric of DSD and DGD), 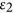, is set to 3.0. The external magnetic field is applied along the z-direction (i.e. the Voigt configuration). Using the end-fire excitation, this configuration can significantly increase the extinction ratio and mimic a momentum matched environment (between surface waves on taper waveguide and on graphene). In the simulation, the TM-polarized plane wave (the electric field is parallel to y-axis) is launched at the beginning of the tapered waveguide (i.e. x = 0) (Fig. 1(a)). The dispersion relation of the surface waves propagating on tapered waveguide in the presence of an applied external magnetic field is also investigated. By suitably choosing the magnitude of applied external magnetic field, this proposed design can efficiently excite the graphene’s surface wave at different chemical potentials (by applying different biased voltages to graphene) and different incident frequencies.

When the external magnetic field is applied onto InSb, its dielectric constant becomes a dielectric tensor whose components depend on the magnitude of magnetic field. Therefore, the optical property of InSb can be manipulated by the applied magnetic field. This is the physical mechanism of magnetic tunability for the proposed structure. The simulated dielectric tensor of InSb under Voigt configuration is expressed as^28^,


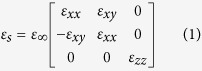


where 
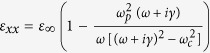
, 
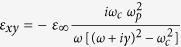
, 
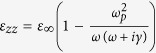
; 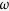 is the angular frequency of incident wave; 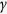, 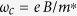 and 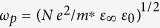 denote the collision frequency, cyclotron frequency and plasma frequency, respectively, where *e*, m* and *N* represent the electron charge, the effective mass and the electron density of free electron, respectively, and *B* is the magnitude of applied external magnetic field; and 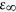
(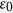) is the high-frequency (vacuum) permittivity. In our simulation, the parameters of InSb at room temperature are set to 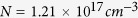, 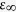 = 15.68, m* = 0.014 *m*_*0*_ (*m*_*0*_ is the electron mass in vacuo), 
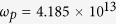
 rad/s and 
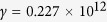
 rad/s^29^. Notably, the collision frequency is assumed a constant in the simulation. The increase of collision caused by electron cyclotron motion has been considered in the dielectric tensor of InSb, Eq. (1).

For modeling the monolayer graphene, the numerical formula for conductivity (from Kubo formula) and relative permittivity are described as below, respectively^30^,^31^,^32^,^33^,^34^,


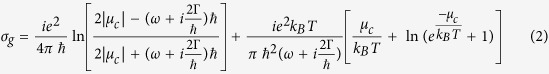



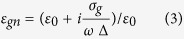


where *e*, 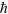 and 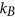 are the electron charge, Plank’s constant and Boltzman’s constant, respectively. In Eq. (2), 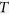
is the temperature in Kelvin, which is fixed to 300 K in this investigation; 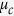 is the chemical potential, which can be electrically controlled by applying biased voltage onto graphene, and hence leads to a voltage-tuned value; 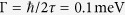, which is related to the electron-phonon coupling loss^31^,^35^. In Eq. (3), 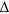 denotes the graphene thickness and is set to 1 nm in this study. (The effective refractive index obtained from 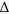 = 1 nm (shown in Fig. 2(a)) is almost the same as that from theoretical calculation in literature^36^). The carrier mobility of graphene used in this work is 51400 cm^2^ V^−1^s^−1^, which is calculated from 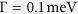 (collision frequency in unit of energy). This value is within the typical range of graphene carrier mobility shown in the literature^37^.

The physical mechanism of efficient excitation of graphene surface wave in the proposed semiconductor tapered waveguide is described as follows. First, the surface waves on the first section of DSD are effectively excited by a plane wave normally incident upon the end face of tapered waveguide (i.e. the end-fire method). (In our simulation, the plane wave will be placed at the beginning of tapered waveguide, i.e. x = 0). This effective excitation of surface wave is ascribed to a much smaller wavevector mismatch between incident light and surface wave. Second, the surface waves propagate in the tapered DSD with wavelength compression due to the gradual increase of their wavevectors. Since InSb is a lossy material (the loss is modeled by the collision frequency in the simulation), the decay of surface waves on the tapered DGD is inevitable. Finally, the graphene surface waves on DGD are efficiently excited at the interface between the fifth section of DSD and DGD because of their wavevectors close to each other. The magnetic tunability of the tapered DSD stems from the fact that the applied magnetic field can manipulate DSD’s dispersion curve.

## Results

Figure 2(a) presents the dispersion curves of light in free space and surface wave on DGD with chemical potential of graphene 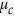 = 0.1 eV^17^. Figure 2(a) exhibits that the difference of wavevectors between light line (solid red line) and surface wave on DGD (dashed green line) is quite large. Note that, the difference of wavevector at the same operating frequency becomes even larger as the value of 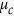 decreases, as shown in Fig. 3(a). Inset of Fig. 2(a) plots the simulated real part of z-component magnetic field (Hz) contours of surface wave on DGD at angular frequency of 26.8 × 10^12^ rad/s, which is excited directly by the end-fired method. In the simulation, the TM-polarized plane wave (the electric field is parallel to y-axis) is launched at the beginning of DGD structure (i.e. x = 0). However, the coupling efficiency is only 5.53%. (The method to determine coupling efficiency is listed in Methods).

**Figure 1:**
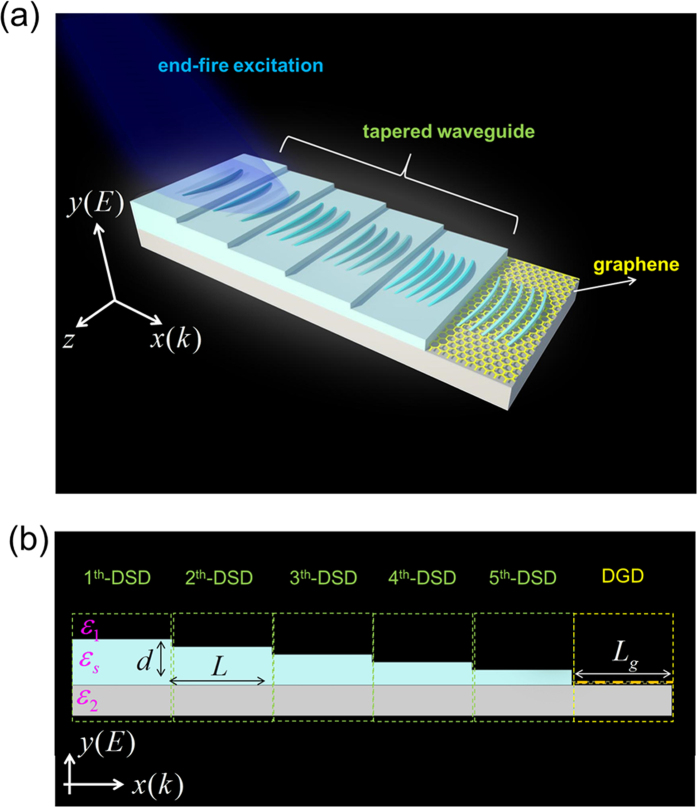
Concept for proposed structure. Three-dimensional (**a**) and side-view (**b**) diagrams of proposed tapered waveguide for efficient excitation of surface wave (surface plasmon) on dielectric-graphene-dielectric (DGD) structure. The tapered waveguide consists of five dielectric-semiconductor-dielectric (DSD) sections. The lower (middle, upper) part of DSD is substrate (semiconductor, air) with relative permittivity of 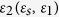. The semiconductor material is InSb. 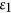 and 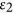 are set to 1.0 and 3.0, respectively. The thicknesses of semiconductor layer (*d*) in five DSD sections are 300, 200, 150, 100, and 80 nm, respectively. The length of each DSD section (*L*) is 5 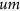. The length of DGD (*L*_*g*_) is 10 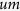.

**Figure 2:**
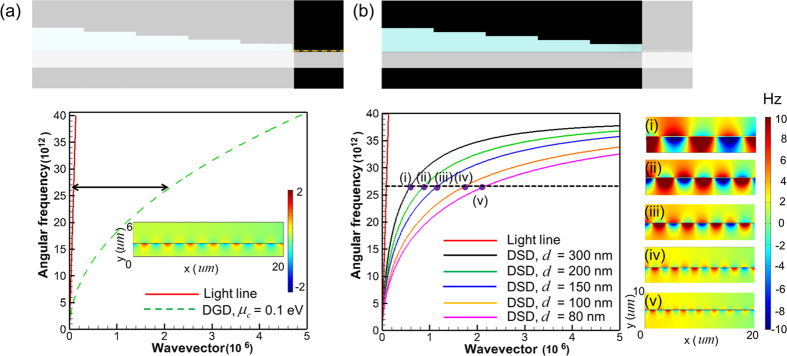
Dispersion curves and real part of Hz field contours. (**a**) Calculated dispersion curves of light in free space (red solid line) and surface wave on DGD with chemical potential 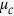 = 0.1 eV (green dashed line). Inset: Simulated real part of Hz field contours in DGD structure at incident frequency of 26.8 × 10^12^ rad/s. The upper schematic diagram (the opaque region) indicates the investigated DGD geometry. (**b**) Dispersion curves of light in free space (red solid line) and surface waves on DSD (all other solid lines) with various *d*’s values. Insets (i), (ii), (iii), (iv), and (v): Simulated real part of Hz field contours in DSD structures with *d* = 300, 200, 150, 100, and 80 nm, respectively, at 26.8 × 10^12^ rad/s. The opaque region in the upper schematic diagram displays the investigated DSD geometry.

Figure 2(b) plots the dispersion curves of DSD structures (which are the components of tapered waveguide) for various thicknesses of InSb layer (*d*). In Fig. 2(b), the black dashed line indicates the incident frequency. (The dispersion relation is derived in Methods). Figure 2(b) displays that, for a fixed incident frequency, the wavelength of excited surface wave decreases with the reduction of *d*, as shown in insets (i)~(v) of Fig. 2(b) (the incident TM-polarized plane wave is also placed at the beginning of DSD structure). It implies that using several DSD structures with gradually decreasing *d* in front of graphene can effectively reduce the wavevector mismatch for exciting surface wave on graphene. Furthermore, since the thickness of InSb layer is larger than that of monolayer graphene, the extinction cross section of the DSD-graphene composite structure also increases. After the surface wave is excited at position of x = 0 (shown in Fig. 1(a)), its wavelength is gradually reduced as it propagates along the taper waveguide (x-direction) while the coupling efficiency become larger than that by directly exciting on the monolayer graphene. In order to facilitate comparison with the literature, the dispersion curves in Fig. 2 are also plotted in the form of wavenumbers (*cm*^−1^) vs. momentum *(k*/*k*_*0*_). (See Figure S1 in the supplementary information; *k*_*0*_ is the wavevector in free space). The propagation lengths of surface waves (defined as the lengths after which the intensities decrease to 1/e) on DGD and DSD at incident frequency of 26.8 × 10^12^ rad/s are also calculated. Based on Fig. 2, the propagation lengths on DGD and DSD with *d* = 300 nm (200 nm, 150 nm, 100 nm, 80 nm) are 25.9 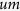 and 45.4 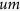 (38.8 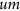, 29.4 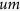, 14.0 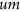, 8.6 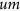), respectively. Notably, the propagation length of surface wave in each section of DSD is larger than the section length (5 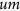). Therefore, the loss in tapered waveguide does not severely affect the coupling efficiency.

**Figure 3:**
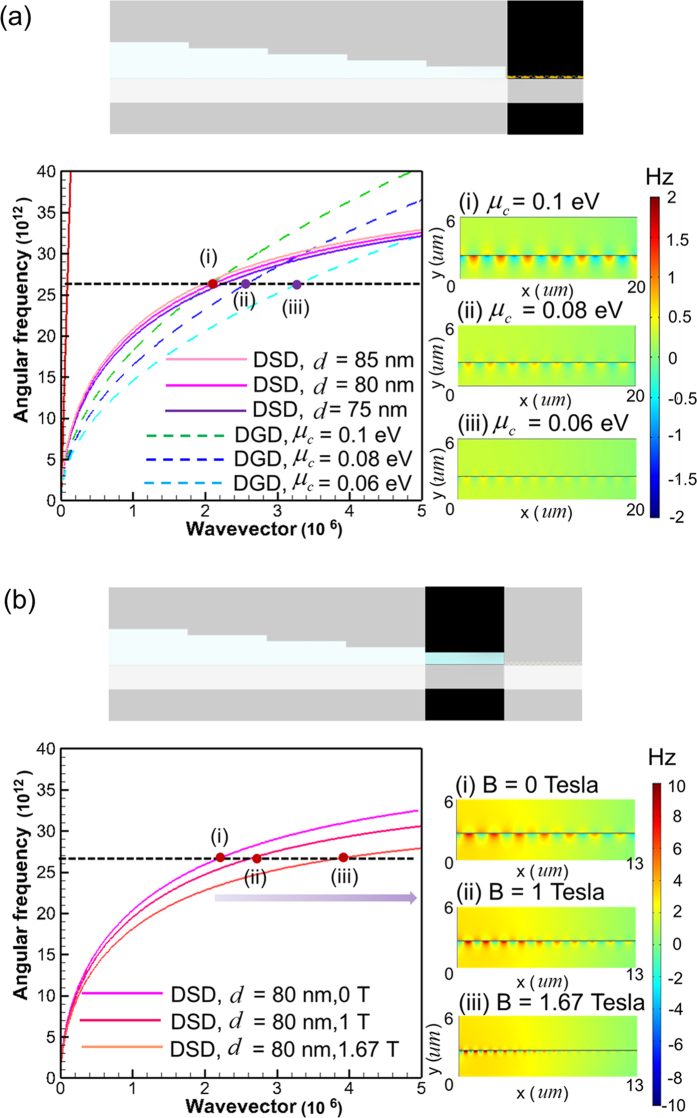
Dispersion curves and real part of Hz field contours. (**a**) Calculated dispersion curves of surface waves on DGD with various 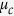
’s values (dash lines) and on DSD with various *d*’s values (solid lines). Insets (i), (ii), and (iii): Simulated real part of Hz field contours in DGD structure with 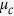 = 0.1, 0.08, and 0.06 eV, respectively, at 26.8 × 10^12^ rad/s. The red solid line represents the dispersion curve of light in free space. (**b**) Calculated dispersion curves of surface waves on DSD structure with applying various *B*’s (external magnetic field) values. Insets (i), (ii), and (iii): Simulated real part of Hz field contours in DSD structure with *B* = 0, 1, and 1.67 Tesla, respectively, at 26.8 × 10^12^ rad/s. The upper schematic diagrams (the opaque region) in (**a**,**b**) show the investigated DGD and DSD structures, respectively.

For improving the feasibility and dynamical reconfigurability of the proposed tapered waveguide, the effects of changing key parameters (such as chemical potential of graphene 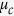, thickness of semiconductor layer *d* and magnitude of applied external magnetic field *B*) on the dispersion curves of DGD and DSD structures are also investigated. Figure 3(a) plots the dispersion curves of DSD and DGD with various values of *d* and 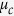, respectively. Figure 3(a) shows that the dispersion curves of DGD with 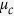 = 0.1 eV and DGD with *d* = 80 nm intersect each other (the red circle symbol in Fig. 3(a)) at 26.8 × 10^12^ rad/s. Therefore, the surface wave on graphene with 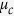 = 0.1 eV can be excited by using a DSD with *d* = 80 nm at this frequency. Figure 3(a) also exhibits that, at the same incident frequency, the wavevectors of surface wave on DSD increase with the reduction of *d*. Worth to mention that, the values of 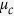 can be modulated by changing the external biased voltage applied onto graphene^16^,^17^,^38^. Insets (i), (ii), and (iii) of Fig. 3(a) present the simulated real part of Hz field contours of DGD with different values of 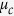 at incident frequency of 26.8 × 10^12^ rad/s. They reveal that the surface waves on graphene become more difficult to be directly excited by end-fire method (i.e. the coupling efficiency becomes smaller) as 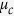 decreases. These results are ascribed to that the reduction in 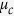 leads to the increase in difference between wavevectors of DGD and light, as shown in Fig. 3 (a).

**Figure 4:**
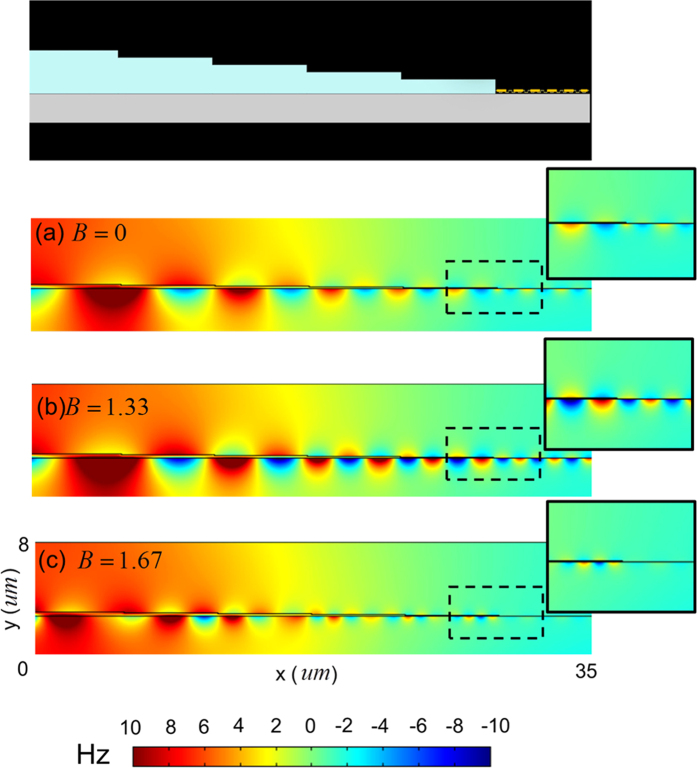
Real part of Hz field contours. Simulated real part of Hz field contour of tapered DSD-DGD composite structure with various values of B for incident frequency of 26.8 × 10^12^ rad/s and 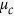 = 0.06 eV. (**a**) *B* = 0 Tesla (**b**) *B* = 1.33 Tesla (**c**) *B* = 1.67 Tesla. Insets: enlarged real part of Hz field contour in black-dashed line region (i.e. around the DSD/DGD area).

Figure 3(b) plots the dispersion curves of DSD under various values of *B* with *d* = 80 nm. (*B* is along the z-direction, i.e. the Voigt configuration). The dispersion relation of DSD with an applied magnetic field under Voigt configuration is also derived in Methods. Figure 3(b) displays that, for a fixed incident frequency, the wavevector of excited surface wave on DSD increases with the magnitude of *B*. Insets (i), (ii), and (iii) of Fig. 3(b) present real part of Hz field contours of DSD with *B* = 0, 1, and 1.67 Tesla, respectively, at incident frequency of 28.6 × 10^12^ rad/s. They confirm that the surface waves can propagate on DSD structure and the wavelength decreases with the increase of *B*’s magnitude. Comparing Fig. 3(ab) indicates that, DSD with *B* = 0 is suitable as a waveguide connector to efficiently excite the surface wave on graphene with its 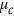 equal to 0.1 eV at frequency of 28.6 × 10^12^ rad/s. When 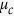 in graphene layer is reduced to 0.06 eV, the surface wave on graphene at the same frequency can be excited by using the same DSD waveguide but with the magnitude of *B* between 1 and 1.67 Tesla. Figure 4 presents the simulated real part of Hz field contours of tapered DSD-DGD composite structure for *B*’s values equal to 0, 1.33 and 1.67 Tesla with incident frequency of 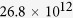 rad/s and 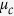 = 0.06 eV. (The coupling efficiencies for Fig. 4(a–c) are 7%, 52% and < 1%, respectively). Figure 4 reveals that the intensity of excited surface wave (the coupling efficiency) reaches a maximum at *B* = 1.33 Tesla. This result is ascribed to that the dispersion curves of surface waves on DGD with 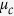 = 0.06 eV and on the fifth section of DSD with *B* = 1.33 Tesla are very close to each other at this incident frequency (see Fig. 3(a,b)). Therefore, efficient excitation of surface waves on DGD with changeable 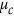 can be attained by using the proposed DSD waveguides with applying an appropriate magnitude of magnetic field onto it. Notably, the external magnetic field considered in this work has little effects on DGD’s dispersion curves because of atomically thin graphene monolayer.

**Figure 5:**
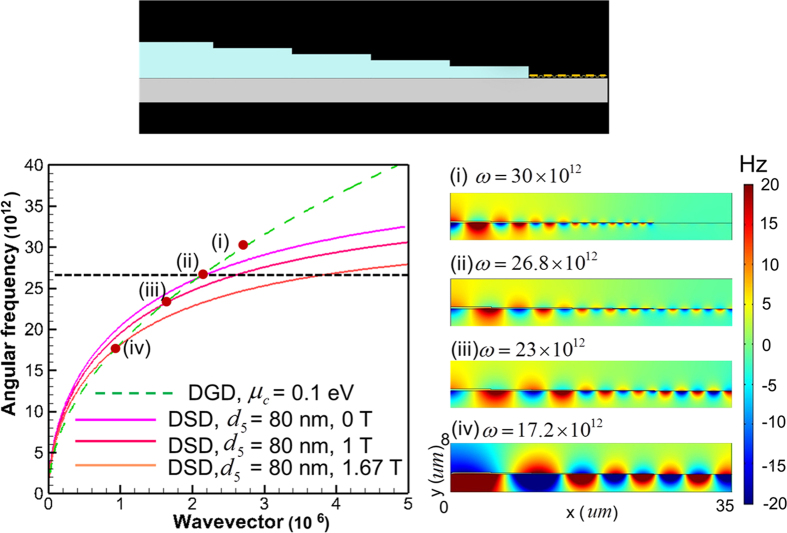
Calculated dispersion curves for surface waves on DSD with various *B*’s values (solid lines) and on DGD with *μ*_*c*_ = 0.1 eV (dashed line). The thickness of semiconductor layer in DSD is 80 nm. Insets (i), (ii), (iii), and (iv): Simulated real part of Hz field contours for excited surface waves on tapered DSD-DGD composite structure at incident frequencies of 30, 26.8, 23 and 17.2 × 10^12^ rad/s, respectively. The upper diagram shows the investigated tapered DSD-DGD composite structure.

Finally, the frequency tunability of surface wave on DGD that is excited by using the proposed tapered DSD waveguide is examined. Figure 5 plots the dispersion curves of DGD (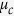 = 0.1 eV) and DSD (*B* = 0, 1 and 1.67 Tesla). Here the DSD structure is the same as the fifth section of the proposed tapered waveguide (*d*_*5*_ = 80 nm, the 5th-DSD in Fig. 1(b)). Figure 5 indicates that the frequency of the intersection point of the dispersion curves decreases with the increase of *B*’s value. As a result, applying an external magnetic field on DSD can extend the operating frequency over a broad range. Furthermore, Fig. 5 also implies that surface wave of 30 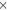
10^12^ rad/s cannot be excited by using the tapered waveguide because of no intersection between the dispersion curves (symbol (i) in Fig. 5). Insets (i)–(iv) of Fig. 5 present the simulated real part of Hz field distributions on tapered DSD-DGD composite structures with incident frequencies of 30, 26.8, 23, 17.2 × 10^12^ rad/s, respectively. They show that, except for 30 × 10^12^ rad/s, the surface waves with all other frequencies are efficiently excited on DGD. The surface waves of 30 × 10^12^ rad/s are only excited in the DSD region. All the results agree with the prediction of dispersion curves in Fig. 5. As we have mentioned, the coupling efficiency for surface wave on DGD by direct end-fire excitation at 26.8 × 10^12^ rad/s is very small (5.53%) due to momentum mismatch between surface wave on DGD and light line. However, when the tapered waveguide is adopted, the coupling efficiency at the same frequency increases to 68.7% (insets (ii) of Fig. 5, *B* = 0). For 23 and 17.2 × 10^12^ rad/s with *B* = 1 and 1.67 Tesla, respectively, the coupling efficiencies of surface waves on DGD are also substantially improved (insets (iii) and (iv) of Fig. 5). The intersection of dispersion curves in Fig. 5 also reveals that the surface waves on DGD and DSD have the same wavelengths at the frequencies of interception points. The simulation results shown in insets (ii), (iii), and (iv) of Fig. 5 are in agreement with what the dispersion curves show. The frequency manipulation of the proposed tapered DSD structure can also be achieved by tuning the constituent parameters 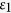, 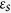, 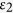 and *d* of DSD structure^39^. However, changing the constituent parameters of DSD is very inconvenient in practical applications. Here we demonstrate that the frequency manipulation in the proposed structure can be accomplished by only tuning magnitude of the applied magnetic field.

The dispersion curves of surface wave on DGD with 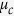 between 0.1 eV and 0.5 eV are also calculated. These dispersion curves show that the difference of wavevectors between light and surface wave on DGD decreases as the value of chemical potential increases (see Supplementary information, Fig. S2). Here the simulation results have demonstrated that the proposed structure can efficiently excite the surface waves on DGD with 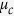 = 0.1 eV in graphene. Then, the surface waves on DGD with larger 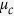 in graphene (e.g. 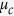 = 0.5 eV) can also be efficiently excited by using a similar structure.

In this work, parylene thin film can be used as the substrate in our proposed design. Its relative permittivity is close to 3.0 at the operating frequencies^40^. Parylene thin film will be deposited on InSb wafer by using the chemical vacuum deposition method first. Then, the tapered structure with various thicknesses can be fabricated on the other side of InSb wafer by using the electron beam lithography and followed by the reactive ion etching to remove unneeded InSb. How the substrate material affects the surface waves on DGD is also considered. The dispersion curves of surface waves on DGD with various values of 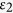 are calculated. They reveal that the difference of wavevectors between light and surface wave on DGD decreases as the value of 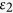 decreases (see Supplementary information, Fig. S3).

## Discussion

In summary, the DSD tapered waveguide with magnetic tunability for efficient excitation of surface waves on DGD at infrared region is proposed and analyzed. Excitation of surface waves on DGD with various chemical potentials in graphene layer and incident frequencies can be attained by merely changing the values of external magnetic field applied onto the DSD tapered waveguide. The FEM electromagnetic simulations verify the design of the proposed structure. More importantly, the constituent materials used in the proposed structure are available in nature. Hence, our methodology can be applied in the field of use of surface waves, such as multi-functional material (device), real-time subwavelength imaging, and high-density optoelectronic components. It will provide us a path to overcome the major issue of energy dissipation of surface waves in conventional metallic material (such as gold, aluminum, and silver).

## Methods

All the simulated results shown in Figs 2, 3, 4, 5 are obtained by using commercial electromagnetic software COMSOL Multiphysics based on the finite element method (FEM). The perfectly matched layers (PML) are used in all propagating directions.

The dispersion relation of surface wave on each three-layered DSD structure without applying the external magnetic field is described as,


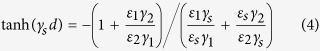


where 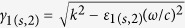 is the wavevector perpendicular to the interface in the upper (middle, lower) medium with the relative permittivity of 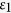 (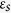, and 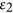).

When the external magnetic field is applied onto the DSD structure, the dispersion relation is transformed into the following form:


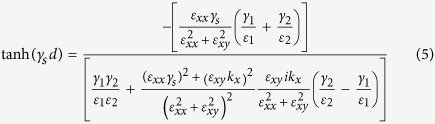


where 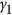, 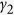 and 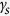 are the same as those in Eq. (4) except for 
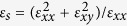
, which is the Voigt dielectric constant. (The derivation of dispersion relation is given in Supplementary information, 4).

The coupling efficiency of excitation of surface wave on DGD is defined as 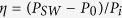, where 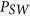 is the time-averaged Poynting power of the surface wave on DGD; 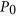 is the same time-averaged Poynting power except that the graphene layer is removed (i.e. only the substrate is left in the simulation); and 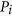 represents the time-average Poynting power at the starting position of the tapered waveguide (i.e., the power that enters the tapered waveguide). 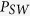, 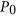 and 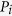 are all acquired from the simulated field distribution contours.

## Additional Information

**How to cite this article**: Cheng, B. H. *et al*. Tunable tapered waveguide for efficient compression of light to graphene surface plasmons. *Sci. Rep.* **6**, 28799; doi: 10.1038/srep28799 (2016).

## Supplementary Material

Supplementary Information

## References

[b1] Barnes W. L., Dereux A. & Ebbesen T. W. Surface plasmon subwavelength optics. Nature 424, 824–830 (2003). 12917696 10.1038/nature01937

[b2] Jacob Z., Alekseyev L. V. & Narimanov E. Optical hyperlens: far-field imaging beyond the diffraction limit. Opt. Express 14, 8247–8256 (2006). 19529199 10.1364/oe.14.008247

[b3] Liu Z., Lee H., Xiong Y., Sun C. & Zhang X. Far-field optical hyperlens magnifying sub-diffraction-limited objects. Science 315, 1686 (2007). 17379801 10.1126/science.1137368

[b4] Cheng B. H., Ho Y. Z., Lan Y. C. & Tsai D. P. Optical hybrid-superlens-hyperlens for superresolution imaging. IEEE J. Sel. Top. Quantum Electron. 19, 4601305 (2013).

[b5] Cheng B. H., Chang K. J., Lan Y. C. & Tsai D. P. Achieving planar plasmonic subwavelength resolution using alternately arranged isulator-metal and insulator-insulator-metal composite structures. Sci. Rep. 5, 7996 (2015). 25613463 10.1038/srep07996 PMC4303896

[b6] Hsu W. L. . Vertical split-ring resonator based anomalous beam steering with high extinction ratio. Sci. Rep. 5, 11226 (2015). 26054048 10.1038/srep11226 PMC4459221

[b7] Kabashin A. V. . Plasmonic nanorod metamaterials for biosensing. Nat. Mater. 8, 867–871 (2009). 19820701 10.1038/nmat2546

[b8] Veselago V. G. The electrodynamics of substances with simultaneously negative values of ε and μ. Sov. Phys.Usp. 10, 509–514 (1968).

[b9] Maier S. Plasmonics: Fundamentals and Applications 1st edn (Springer Verlag, 2007).

[b10] Yu N. . Light Propagation with Phase Discontinuities: Generalized Laws of Reflection and Refraction. Science 334, 333–337 (2011). 21885733 10.1126/science.1210713

[b11] Kildishev A. V., Boltasseva A. & Shalaev V. M. Planar photonics with metasurfaces. Science 339, 1232009 (2013). 23493714 10.1126/science.1232009

[b12] Litchinitser N. M. Applied Physics. Structured Light Meets Structured Matter. Science 37, 1054–1055 (2012). 22936768 10.1126/science.1226204

[b13] Sun S. L. . Gradient-index meta-surfaces as a bridge linking propagating waves and surface waves. Nat. Mater. 11, 426–431 (2012). 22466746 10.1038/nmat3292

[b14] Linic S., Christopher P. & Ingram D. B. Plasmonic-metal nanostructures for efficient conversion of solar to chemical energy. Nature Mater. 10, 911–921 (2011). 22109608 10.1038/nmat3151

[b15] Zhang X., Chen Y. L., Liu R. S. & Tsai D. P. Plasmonic photocatalysis. Reports on Progress in Physics 76, 046401 (2013). 23455654 10.1088/0034-4885/76/4/046401

[b16] Ozbay E. Plasmonics Merging photonics and electronics at nanoscale dimensions. Science 311, 189–193 (2006). 16410515 10.1126/science.1114849

[b17] Vakil A. & Engheta N. Transformation optics using graphene. Science 332, 1291–1294 (2011). 21659598 10.1126/science.1202691

[b18] Fan Y., Shen N.-H., Koschny T. & Soukoulis C. M. Tunable terahertz meta-surface with graphene cut-wires. ACS Photonics 2, 151–156 (2015).

[b19] Soto Lamata I., Alonso-Gonzalez P., Hillenbrand R. & Nikitin A. Y. Plasmons in Cylindrical 2D Materials as a Platform for Nanophotonic Circuits. ACS Photonics 2, 280–286 (2015).

[b20] Grigorenko A. N., Polini M. & Novoselov K. S. Graphene plasmonics. Nat. photonics 6, 749–758 (2012).

[b21] Gao W. . Excitation and active control of propagating surface plasmon polaritons in graphene. Nano Lett. 13, 3698–3702 (2013). 23895501 10.1021/nl401591k

[b22] Yu R., Pruneri V. & de Abajo F. J. G. Resonant visible light modulation with graphene. ACS Photonics 2, 550–558 (2015).

[b23] Nikitin A. Y., Alonso-González P. & Hillenbrand R. Efficient Coupling of Light to Graphene Plasmons by Compressing Surface Polaritons with Tapered Bulk Materials. Nano Lett. 14, 2896–2901 (2014). 24773123 10.1021/nl500943r

[b24] Zhao T. . Coherent and Tunable Terahertz Radiation from Graphene Surface Plasmon Polarirons Excited by Cyclotron Electron Beam. Sci. Rep. 5, 16059 (2015). 26525516 10.1038/srep16059 PMC4630615

[b25] Chen J. . Strong plasmon reflection at nanometer-size gaps in monolayer graphene on SiC. Nano Lett. 13, 6210–6215 (2013). 24188400 10.1021/nl403622t

[b26] Andryiesuski A. & Lavrinenko A. V. Nanocouplers for infrared and visible light. Adv. Optoelectron. 2012, 839747 (2012).

[b27] Agio M., Chen X. W. & Sandoghdar V. Nanofocusing radially-polarized beams for high-throughput funneling of optical energy to the near field. Opt. Express 18, 10878–10887 (2010). 20588943 10.1364/OE.18.010878

[b28] Brion J. J., Wallis R. F., Hartstein A. & Burstein E. Theory of surface magnetoplasmons in semiconductors. Phys. Rev. Lett. 28, 1455–1458 (1972).

[b29] Wu H. H. & Lan Y. C. Magnetic lenses of surface magnetoplasmons in semiconductor–glass waveguide arrays. Appl. Phys. Express 7, 032203 (2014).

[b30] Amet F. . Composite fermions and broken symmetries in graphene. Nat. Commun. 6, 5838 (2015). 25562690 10.1038/ncomms6838

[b31] Hanson G. W. Dyadic Greens functions and guided surface waves on graphene. J. Appl. Phys. 103, 064302 (2006).

[b32] Falkovsky L. A. & Pershoguba S. S. Optical Far-Infrared Properties of a Graphene Monolayer and Multilayer. Phys. Rev. B 76, 153410 (2007).

[b33] Hwang E. H. & Das Sarma S. Dielectric function, screening, and plasmons in two-dimensional graphene. Phys. Rev. B 75, 205418 (2007).

[b34] Gusynin V. P., Sharapov S. G. & Carbotte J. P. Magneto-optical conductivity in graphene. J. Phys. Condens. Matter 19, 026222 (2007).

[b35] Jishi R. A., Dresselhaus M. S. & Dresselhaus G. Electron-phonon coupling and the electrical conductivity of fullerene nanotubules. Phys. Rev. B 48, 11385–11389 (1993). 10.1103/physrevb.48.11385 10007453

[b36] Alaee R., Farhat M., Rockstuhl C. & Lederer F. A perfect absorber made of a graphene micro-ribbon metamaterial. Optics Express 20, 28017–28024 (2012). 23263036 10.1364/OE.20.028017

[b37] Gao W., Shu J., Qiu C. & Xu Q. Excitation of plasmonic waves in graphene by guided-mode resonances. ACS Nano 6, 7806–7813 (2012). 22862147 10.1021/nn301888e

[b38] Cheng B. H., Chang K. J., Lan Y. C. & Tsai D. P. Actively controlled super-resolution using graphene-based structure. Optics Express 22, 28635–28644 (2014). 25402104 10.1364/OE.22.028635

[b39] Karalis A., Lidorikis E., Ibanescu M., Joannopoulos J. D. & Soljacić M. Surface-plasmon-assisted guiding of broadband slow and subwavelength light in air. Phys. Rev. Lett. 95, 063901 (2005). 16090954 10.1103/PhysRevLett.95.063901

[b40] Liu X. . Metamaterials on parylene thin film substrates: Design, fabrication, and characterization at terahertz frequency. App. Phys. Lett. 96, 011906 (2010).

