# Supplementary material for: Tunable tapered waveguide for efficient compression of light to graphene surface plasmons
Source: Sci Rep. 2016 Jun 29;6:28799. doi: 10.1038/srep28799 (PMC4926281; doi:10.1038/srep28799)
Supplement: Supplementary Information [file srep28799-s1.doc]

Supplementary Information

**Tunable tapered waveguide for efficient compression of light to graphene surface plasmons**

*Bo Han Cheng, Hong Wen Chen, Yi-Jun Jen, Yung-Chiang Lan, and Din Ping Tsai*

**1. Dispersion curves of surface waves of Fig. 2 in the form of *cm^-1^* vs. *k/k_0_***


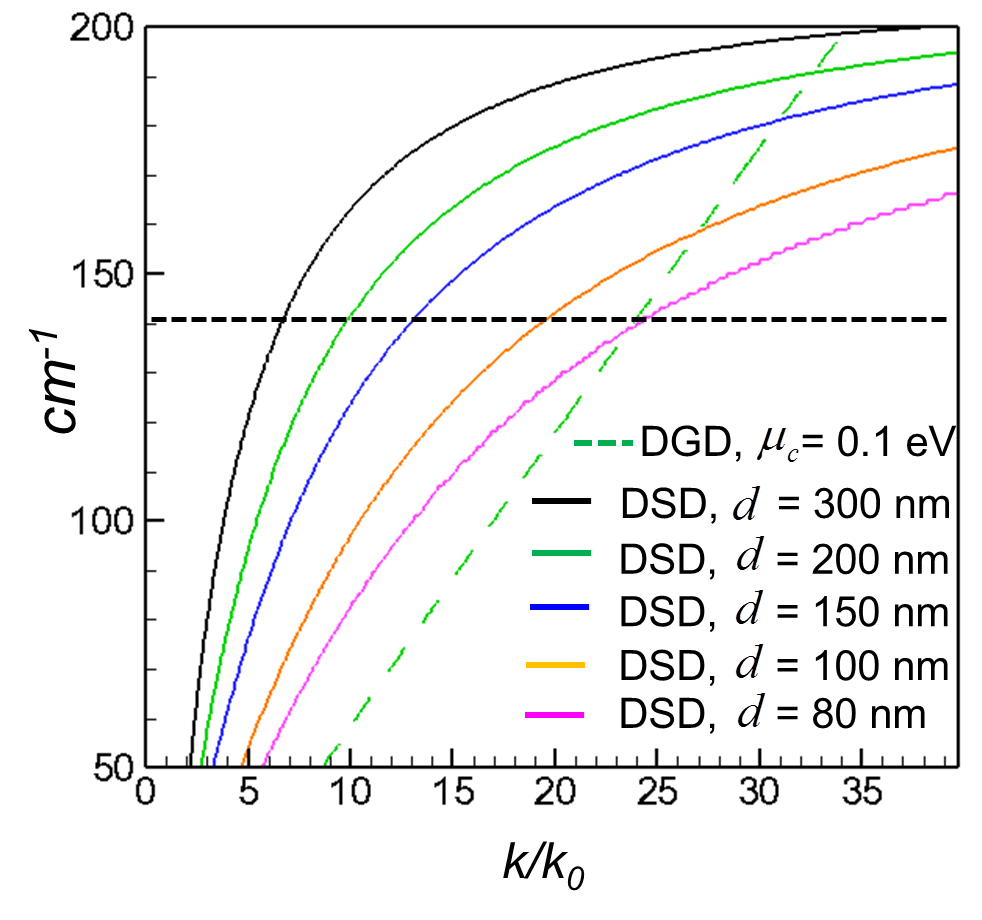


**Figure S1** Calculated dispersion curves (*cm^-1^* vs. *k/k_0_*) of surface waves on DGD with chemical potential $\mu_{c}$= 0.1 eV (green dashed line) and on DSD with various *d*’s values (all other solid lines).

**2. Dispersion curves of surface waves on dielectric-graphene-dielectric (DGD) structure with chemical potentials between 0.1 eV and 0.5 eV**


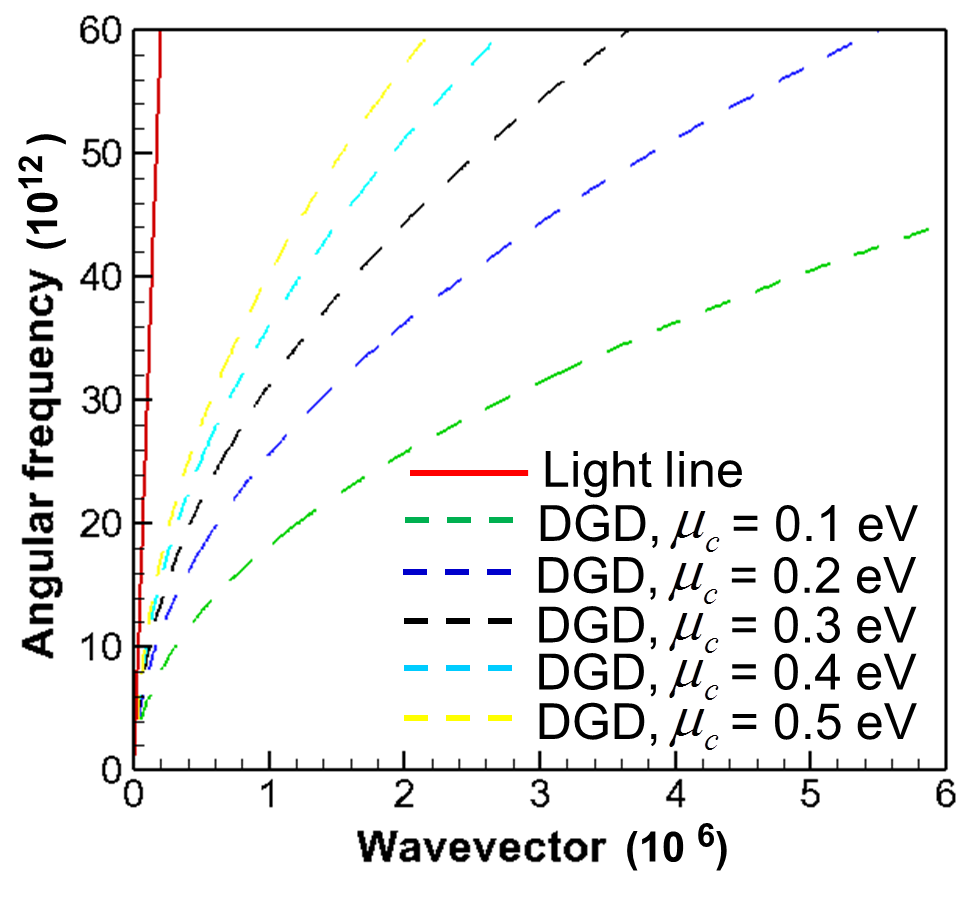


**Figure S2** Calculated dispersion curves of light in free space (red solid line) and surface waves on DGD with various chemical potentials (dashed line). All other parameters for determining the dispersion curves are the same as Figure 2(a).

**3. Dispersion curves of surface waves on dielectric-graphene-dielectric (DGD) structure with various values of** $\varepsilon_{2}$


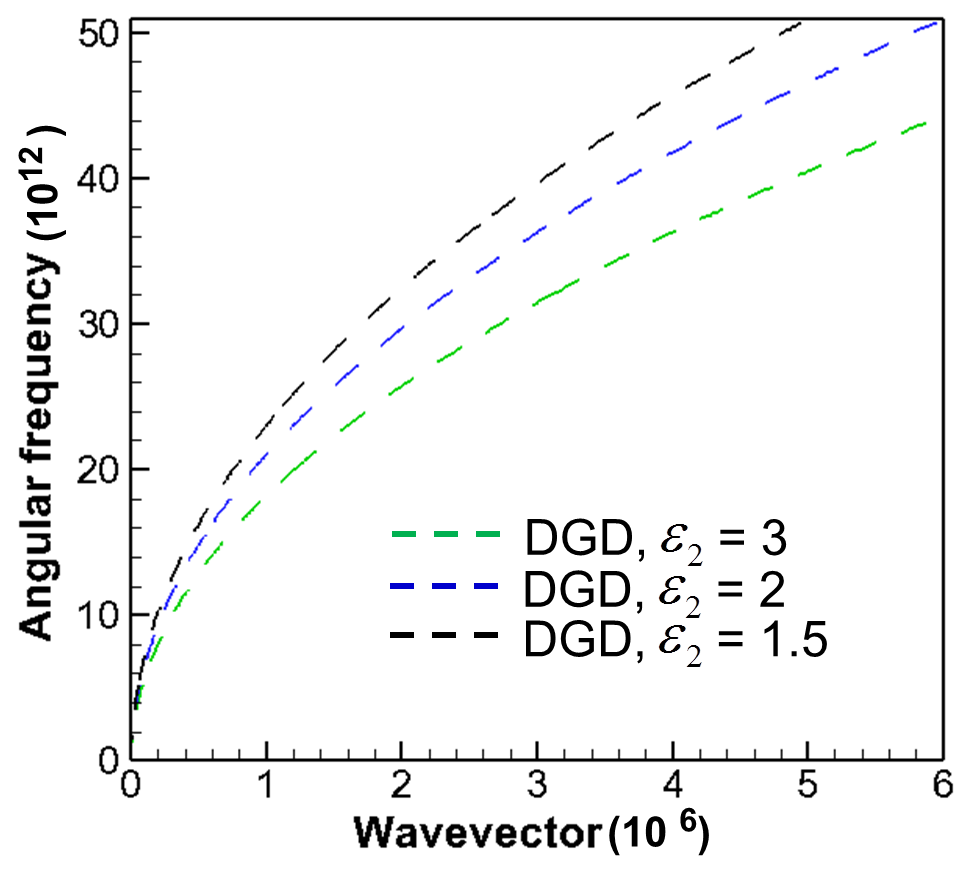


**Figure S3** Calculated dispersion curves of surface wave on DGD with various values of $\varepsilon_{2}$ (dashed line). All other parameters for determining dispersion curve are the same as green dashed line of Figure 2(a).

**4. Derivation of dispersion relation of dielectric-semiconductor-dielectric (DSD) structure with applying external magnetic field onto it**


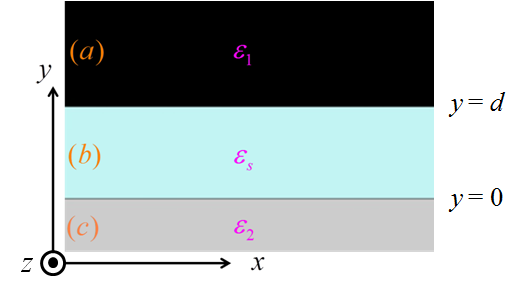


**Figure S4** Schematic of three-layered dielectric-semiconductor-dielectric (DSD) structure under Voigt configuration.

In region (a), (b), and (c), the *H_z_* field can be respectively described by,

(a): $H_{z1}=Ae^{-\gamma_{1}y}$ (1)

(b): $H_{z2}=Be^{-\gamma_{s}y}+Ce^{\gamma_{s}y}$ (2)

(c): $H_{z3}=De^{\gamma_{2}y}$ (3)

where $\gamma_{1}=\sqrt{k^{2}-\varepsilon_{1}\left(\omega/c\right)^{2}},$ $\gamma_{s}=\sqrt{k^{2}-\varepsilon_{s}\left(\omega/c\right)^{2}},$and $\gamma_{2}=\sqrt{k^{2}-\varepsilon_{2}\left(\omega/c\right)^{2}}.$

Note that, $\varepsilon{}_{1}$, $\varepsilon{}_{s}$ and $\varepsilon{}_{2}$ denote the relative permittivities in region (a), (b), and (c), respectively. (Under the Voigt configuration, $\varepsilon_{s}=\left(\varepsilon_{xx}^{2}+\varepsilon_{xy}^{2}\right)/\varepsilon_{xx}$.)

Using the equation $\nabla\times H=-j\omega\varepsilon E,$ the electric field *E_x_* can be expressed as,

(a): $E_{x1}=\frac{\gamma_{1}}{j\omega\varepsilon_{1}}Ae^{-\gamma_{1}y}$ (4)

(b): $E_{x2}=\left(\frac{\varepsilon_{xx}\gamma_{s}-\varepsilon_{xy}ik_{x}}{j\omega\left(\varepsilon_{xx}^{2}+\varepsilon_{xy}^{2}\right)}\right)Be^{-\gamma_{s}y}-\left(\frac{\varepsilon_{xx}\gamma_{s}+\varepsilon_{xy}ik_{x}}{j\omega\left(\varepsilon_{xx}^{2}+\varepsilon_{xy}^{2}\right)}\right)Ce^{\gamma_{s}y}$ (5)

(c): $E_{x3}=\frac{-\gamma_{2}}{j\omega\varepsilon_{2}}De^{\gamma_{2}y}$ (6)

Considering the boundary condition

$$\begin{matrix} H_{z1}(d)=H_{z2}(d) \\ H_{z2}(0)=H_{z3}(0) \\ E_{x1}(d)=E_{x2}(d) \\ E_{x2}(0)=E_{x3}(0) \end{matrix}$$

We can get,

$$\begin{matrix} Ae^{-\gamma_{1}d}=Be^{-\gamma_{s}d}+Ce^{\gamma_{s}d} \\ B+C=D \\ \frac{\gamma_{1}}{\varepsilon_{1}}Ae^{-\gamma_{1}d}=\frac{\varepsilon_{xx}\gamma_{s}-\varepsilon_{xy}ik_{x}}{\left(\varepsilon_{xx}^{2}+\varepsilon_{xy}^{2}\right)}Be^{-\gamma_{s}d}-\frac{\varepsilon_{xx}\gamma_{s}+\varepsilon_{xy}ik_{x}}{\left(\varepsilon_{xx}^{2}+\varepsilon_{xy}^{2}\right)}Ce^{\gamma_{s}d} \\ \frac{\varepsilon_{xx}\gamma_{s}-\varepsilon_{xy}ik_{x}}{\left(\varepsilon_{xx}^{2}+\varepsilon_{xy}^{2}\right)}B-\frac{\varepsilon_{xx}\gamma_{s}+\varepsilon_{xy}ik_{x}}{\left(\varepsilon_{xx}^{2}+\varepsilon_{xy}^{2}\right)}C=\frac{-\gamma_{2}}{\varepsilon_{2}}D \end{matrix}$$

(7)

Reorganizing above equation yields:

$\left[\begin{matrix} \left(\frac{\gamma_{1}}{\varepsilon_{1}}-\frac{\varepsilon_{xx}\gamma_{s}-\varepsilon_{xy}ik_{x}}{\varepsilon_{xx}^{2}+\varepsilon_{xy}^{2}}\right)e^{-\gamma_{s}d} & \left(\frac{\gamma_{1}}{\varepsilon_{1}}-\frac{\varepsilon_{xx}\gamma_{s}+\varepsilon_{xy}ik_{x}}{\varepsilon_{xx}^{2}+\varepsilon_{xy}^{2}}\right)e^{\gamma_{s}d} \\ \frac{\gamma_{2}}{\varepsilon_{2}}-\frac{\varepsilon_{xx}\gamma_{s}-\varepsilon_{xy}ik_{x}}{\varepsilon_{xx}^{2}+\varepsilon_{xy}^{2}} & \frac{\gamma_{2}}{\varepsilon_{2}}-\frac{\varepsilon_{xx}\gamma_{s}+\varepsilon_{xy}ik_{x}}{\varepsilon_{xx}^{2}+\varepsilon_{xy}^{2}} \end{matrix}\right]\left[\begin{matrix} B \\ C \end{matrix}\right]=0$ (8)

Finally, solving this system of equations results in the following dispersion relation formula,

$\tanh(\gamma_{s}d)=\frac{-\left[\frac{\varepsilon_{xx}\gamma_{s}}{\varepsilon_{xx}^{2}+\varepsilon_{xy}^{2}}\left(\frac{\gamma_{1}}{\varepsilon_{1}}+\frac{\gamma_{2}}{\varepsilon_{2}}\right)\right]}{\left[\frac{\gamma_{1}\gamma_{2}}{\varepsilon_{1}\varepsilon_{2}}+\frac{\left(\varepsilon_{xx}\gamma_{s}\right)^{2}+\left(\varepsilon_{xy}k_{x}\right)^{2}}{\left(\varepsilon_{xx}^{2}+\varepsilon_{xy}^{2}\right)^{2}}\frac{\varepsilon_{xy}ik_{x}}{\varepsilon_{xx}^{2}+\varepsilon_{xy}^{2}}\left(\frac{\gamma_{2}}{\varepsilon_{2}}-\frac{\gamma_{1}}{\varepsilon_{1}}\right)\right]}$ (9)
